# The PPR-Domain Protein SOAR1 Regulates Salt Tolerance in Rice

**DOI:** 10.1186/s12284-022-00608-x

**Published:** 2022-12-03

**Authors:** Kai Lu, Cheng Li, Ju Guan, Wen-Hua Liang, Tao Chen, Qing-Yong Zhao, Zhen Zhu, Shu Yao, Lei He, Xiao-Dong Wei, Ling Zhao, Li-Hui Zhou, Chun-Fang Zhao, Cai-Lin Wang, Ya-Dong Zhang

**Affiliations:** Institute of Food Crops, Jiangsu Academy of Agricultural Sciences, National Center of Technology Innovation for Saline-Alkali Tolerant Rice, Jiangsu High Quality Rice Research and Development Center, Nanjing Branch of China National Center for Rice Improvement, 210014 Nanjing, China

**Keywords:** Salt stress, Rice, Pentatricopeptide repeat (PPR) protein, SOAR1, Alternative splicing

## Abstract

Previous studies in *Arabidopsis* reported that the PPR protein SOAR1 plays critical roles in plant response to salt stress. In this study, we reported that expression of the *Arabidopsis* SOAR1 (AtSOAR1) in rice significantly enhanced salt tolerance at seedling growth stage and promoted grain productivity under salt stress without affecting plant productivity under non-stressful conditions. The transgenic rice lines expressing AtSOAR1 exhibited increased ABA sensitivity in ABA-induced inhibition of seedling growth, and showed altered transcription and splicing of numerous genes associated with salt stress, which may explain salt tolerance of the transgenic plants. Further, we overexpressed the homologous gene of *SOAR1* in rice, *OsSOAR1*, and showed that transgenic plants overexpressing OsSOAR1 enhanced salt tolerance at seedling growth stage. Five salt- and other abiotic stress-induced SOAR1-like *PPRs* were also identified. These data showed that the SOAR1-like PPR proteins are positively involved in plant response to salt stress and may be used for crop improvement in rice under salinity conditions through transgenic manipulation.

**Supplementary Information:**

The online version contains supplementary material available at 10.1186/s12284-022-00608-x.

## Background

Salt stress is one of major adverse environmental stresses that seriously limit plant growth and crop productivity (Zhu et al. 2002; Roy et al. 2011). The main reasons for repressing plant growth and development under salt stress are osmotic stress and Na^+^ toxic, causing difficulty in water uptake, inhibition of cell elongation and leaf development, reducing of enzyme activity and photosynthesis (Hasegawa et al. 2000; Wankhade et al. 2010). Plants continue to evolve in the process of adapting to salt stress, thus forming a variety of stress response mechanisms at the level of gene expression regulation, such as transcriptional, post- transcriptional and epigenetic regulation (Munns et al. 2008; Shang et al. 2017; Saradadevi et al. 2021). Pre-mRNA splicing contributes to cellular gene expression and plants stress responses (Laloum et al. 2018; Yang et al. 2018; Jabre et al. 2019).

Pentatricopeptide repeat (PPR) proteins compose one of the largest protein families in higher plants with 450 and 491 members in *Arabidopsis* and rice respectively, which is featured by the presence of 2–50 times tandem arrays of the PPR motif, a degenerate 35 amino acid repeat structure (Small et al. 2000; Barkan et al. 2014; Chen et al. 2018; Zhang et al. 2019). PPR proteins are considered to be sequence-specific RNA-binding proteins via the recognition of specific nucleotides by the PPR motifs (Yin et al. 2013; Shen et al. 2016). PPR proteins are divided into two classes, P and PLS, according to their characterization and arrangement of PPR motifs (Lurin et al. 2004). The P-class proteins contain tandem arrays of typical P-motifs with 35 amino acids, whereas the PLS-class proteins contain tandemly arrangement of PLS triplets with P-, L-(35–36 amino acids) and S-(31–32 amino acids) motifs which can be further classified into four subgroups, PLS, E, E + and DYW, based on the characteristics of C-terminal domains of PPR proteins (Barkan et al. 2014; Cheng et al. 2016). The PLS-class of PPR proteins mainly function in the C-U editing of mitochondrial RNA and the P-class of PPR proteins are involved in multiple processes of RNA post-transcriptional regulation, such as alternative splicing of pre-mRNA, RNA editing and stability (Barkan et al. 2014; Zhao et al. 2020). Most PPR members have been reported to be localized to the mitochondria or chloroplasts, and fewer PPR proteins are localized to other cellular compartments to regulate RNA processing involved in diverse physiological process, such as seed development, male sterility, and abiotic stress (Hammani et al. 2011; Tang et al. 2014; Li et al. 2021).

Previous studies have reported that PPR proteins were involved in plants response to salt and other abiotic stress (Zsigmond et al. 2008; Murayama et al. 2012; Emami et al. 2020; Ganie and Reddy 2021). Mutation of the mitochondrial electron transporting-linked PPR40 results in enhanced sensitivity to salt, abscisic acid, and oxidative stress because of the accumulation of reactive oxygen species in *Arabidopsis* (Zsigmond et al. 2008). Loss-of-function mutation of the PENTATRICOPEPTIDE REPEAT PROTEIN FOR GERMINATION ON NaCl (PGN) showed hypersensitive response to abscisic acid (ABA), glucose and NaCl, which accumulated higher level of endogenous ABA by elevating expression of ABA biosynthesis gene *NCED3* (Laluk et al. 2011). *Arabidopsis* ABA hypersensitive germination 11 (AHG11) was identified as a mitochondrial PPR protein responsible for editing of *NAD4* transcripts of complex I, both of which showed increased sensitivity to ABA and accumulated reactive oxygen species (Murayama et al. 2012). T-DNA insertion mutant of the salt and oxidative stress induced *PPR* gene, *PPR96*, exhibited enhanced tolerance to salt, ABA and oxidative stress through regulating transcription level of stress-responsive genes under abiotic stress treatment (Liu et al. 2016). The chloroplast-localized PPR proteins White Stripe Leaf (WSL), reported to be necessary for splicing of *rpl2*, affects chloroplast development and is positively involved in plant response to ABA, salinity, and sugar (Tan et al. 2014). The PPR protein NATURAL BLIGHT LEAF 3 (NBL3) was found to be essential for mitochondrial development and functions, which mutation leads to improved tolerance to salt stress and reduced activity of complex I (Qiu et al. 2021). Other PPR proteins were also reported to play important roles in plant response to cold stress, oxidative stress and ABA signaling (Liu et al. 2010; Yuan et al. 2012; Gong et al. 2014; Wu et al. 2016, 2018; Emami et al. 2020).

It was reported that the transgenic *Arabidopsis* plants of the cytosol- and nucleus-localized PPR protein, SOAR1 (suppressor of the ABAR-overexpressor 1), showed strong tolerance ability to salt, drought and cold stress, especially to high concentration of NaCl proximately to seawater in germination and post-germination growth (Mei et al. 2014; Jiang et al. 2015; Ma et al. 2020). To assess the application value of SOAR1 in crop improvement under salinity conditions, we overexpressed the *AtSOAR1* gene in a widely spread local *japonica* rice cultivar, NanGeng9108, and the results showed that expression of AtSOAR1 in rice significantly enhanced salt tolerance both at seedling growth stage and at total growth period. Further experimental data showed that transgenic plants expression of the homologous gene of *AtSOAR1* in rice, *OsSOAR1*, display salt-tolerance phenotype at seedling growth stage compared with wild-type plants, which suggests that both *AtSOAR1* and *OsSOAR1* were likely to be used for crop improvement under salinity conditions.

## Materials and Methods

### Plant Materials and Growth Conditions

The local *japonica* rice cultivar NanGeng9108 (NG9108) in Jiangsu province of China was used as wild-type control in this work. The locus ids for *AtSOAR1* and *OsSOAR1* were At5g11310 and Os01g0506100, respectively. For the generation of the overexpression plants of *AtSOAR1*, the open reading frame (ORF) sequences of *AtSOAR1* was amplified and constructed into a modified pCAMBIA1300 vector (http://www.cambia.org) with *Flag* tag. For the generation of the overexpression plants of *OsSOAR1*, the ORF sequences of *OsSOAR1* was amplified and constructed into pMDC85 vector (https://www.arabidopsis.org/) with *GFP* tag. Both of the overexpression vectors were driven by cauliflower mosaic virus (CaMV) 35S promoter. The resulting plasmids were introduced into *Agrobacterium tumefaciens* strain GV3101, which was then transformed into wild-type plants by *Agrobacterium*-mediated callus transformation method (Nishimura et al. 2006). Transgenic plants containing single T-DNA insertion were screened by hygromycin resistance and confirmed by real-time quantitative PCR (qPCR). The homozygous lines for overexpressing *AtSOAR1* (OE-1 and OE-2) or *OsSOAR1* (OsSOAR1-OE1 and OsSOAR1-OE2), were used for further analysis. Primer sequences for plasmid construction were listed in Additional file 1: Table S3.

Rice seeds were surface-sterilized and germinated in water solution in a growth chamber (14-h light/10-h dark) with about 300 μmol photons m^−2^ s^−1^ light intensity at 30 °C and 70% relative humidity, and the germinated seeds were then planted in pots which were placed in an isolated experimental field with normal agricultural management.

### Abiotic Stress Treatments and Real-Time PCR Analysis

For treatment of ABA, PEG, drought and cold stress, seeds were germinated in water for 4 d before transferred to liquid 1/2 Murashige and Skoog (MS) hydroponic culture solution and continued to grow for 10 d before treatment. Seedlings were treated with 0 or 10 µM ( ±) ABA, 100 mM PEG, or transferred to a growth chamber at 4 °C for 5 h. For treatment of drought stress, seedlings were cultured without water supply, exposing roots to air for 2 h. The materials were then collected and sampled for RNA extraction. Whole seedlings growing for two weeks under normal growth condition were used for detecting transcript level of *AtSOAR1* or *OsSOAR1* in the wild-type and the corresponding transgenic plants. Seedlings grown in solutions supplemented with different concentration of ( ±) ABA were sampled for determination of ABA-responsive genes transcription in the *AtSAOR1*-transgenic plants compared with wild-type plants. RNA was isolated using a Total RNA Rapid Extraction Kit (BioTeke, Beijing, China) and a RNA Purification Kit (BioTeke, Beijing, China) according to the manufacturer’s instructions, respectively. The first-strand cDNA was synthesized using M-MLV reverse transcriptase Kit (Roche, Mannheim, Germany) and qPCR were conducted using a ABI StepOnePlus PCR machine (Thermofisher, Waltham, USA) with SYBR Green PCR Master Mix (Vazyme, Nanjing, China). Rice *Actin1* was used as an internal control. Primer sequences for qPCR were listed in Additional file 1: Table S3.

### Full-Length Transcriptome Analysis

For obtaining rice materials for RNA sequencing, seedlings of wild-type plants incubated for 14 d in hydroponic culture solution as described above were treated with or without 140 mM NaCl for 2 d. The materials were collected and sampled for RNA isolation. 1 μg total RNA was added for cDNA library construction using cDNA-PCR Sequencing Kit (Oxford Nanopore Technologies, UK). Briefly, full-length cDNA was synthesized first by reverse transcriptase, which were then anchored to both ends of the first-strand cDNA by defined PCR adapters. PCR was performed with LongAmp Taq enzyme and PCR products were ligated with ONT adaptor for purification with Agencourt XP beads following ONT protocol. The constructed library was used for full-length RNA-sequencing with PromethION platform (Oxford Nanopore Technologies, UK) and raw reads length above 500 bp were filtered. Each treatment was repeated for three times.

Data collection and bioinformatics analysis services were provided by Biomarker Technology Company (Beijing, China). Briefly, full-length transcripts were determined by searching for primer at both ends of raw reads followed by mapping to the reference genome in RAP-DB (Rice Annotation Project Database) using mimimap2 software. For structure analysis, the alternative splicing events were identified by the AStalavista tool and transcripts were validated against RAP-DB transcript annotations with gffcompare. For gene functional annotation, multiple databases such as NR (NCBI non-redundant protein sequences), Pfam (Protein family), GO (Gene Ontology) were employed. For differential expression analysis, the FDR(false discovery rate) < 0.05 and fold change ≥ 1.5 was set as the threshold for DEGs (differential expressed genes) identification. For pathway enrichment, KOBAS software was used to test the statistical enrichment of differential expression genes in KEGG pathways. All sequencing raw data have been deposited to NCBI with ID: PRJNA869885.

### Phenotype Analysis

For seed germination assay, about 100 seeds of each genotype plants were disinfected using 10% (v/v) sodium hypochlorite for 10 min and cultured in 1/2 MS solution containing different concentration of NaCl or ( ±) ABA after drying for 1 week at 48 °C to completely breaking dormancy. The seeds were placed in the growth chamber and solutions were refreshed every day. The number of germinated seeds was scored and calculated at the indicated times.

For salt tolerance tests at seedling stage, seeds were germinated in water for 4 d before transferred to liquid 1/2 MS hydroponic culture solution and continued to grow for 10 d before treatment. Seedlings were placed to the same culture solution supplemented with 100 mM NaCl for14d or 140 mM NaCl for 8d, which were then transferred to normal culture solution without salt for one-weak recovery. Solutions were refreshed every three days. The numbers of alive seedlings of wild-type and transgenic plants were counted and survival rates were calculated. All the experiments were repeated three times.

For the measurement of ABA-mediated seedling growth arrest, the sterilized rice seeds were cultured in water solution for 4 days and the germinated seeds with similar growth status continued to grow in 1/2 MS solution containing different concentration of ( ±) ABA for 9 d before investigation. Solutions were refreshed every day.

For salt tolerance tests in whole growth period, two-week-old seedlings of wild-type and transgenic plants overexpressing AtSOAR1, OE-1 and OE-2, were planted to a same pot containing normal soil or 0.15% NaCl (g/g, ratio of salt weight to that of dry soil) pre-treated soil to mimic salt stress. The plants were cultured under common agriculture management and three agricultural characters, plant height, tiller number and grain yield per plant, were counted.

### Measurement of Chlorophyll Content and Ion Leakage

For total chlorophyll content assays, 100 mg fresh leaves of rice seedlings treated with or without NaCl were used. The leaves were quick-freezed in liquid nitrogen and were homogenized. After adding 5 ml chlorophyll extraction buffer containing 80% acetone and 20% distilled water, the samples were kept in dark for 1 h at room temperature before being centrifuged at 12,000 g for 3 min. Absorbance of the supernatant at 645 and 663 nm wave length was measured respectively, and the total chlorophyll content was determined according to the following formula: Total chlorophyll (μg/ml) = 20.2 (A645) + 8.02 (A663).

For ion leakage assays, 100 mg fresh leaves of rice seedlings treated with 100 mM NaCl for 24 h were used. The leaves were vacuum infiltrated in deionized water for 20 min and then kept at room temperature for 2 h. Conductivities (C1) of the resulting solution was measured. The samples were boiled for 15 min and cooled to room temperature before being determined for conductivities (C2). The relative ion leakage was determined according to the following formula: Relative ion leakage = C1/C2.

## Results

### Expression of AtSOAR1 in Rice Enhances Salt Tolerance at Seedling Growth Stage

**Fig. 1:**
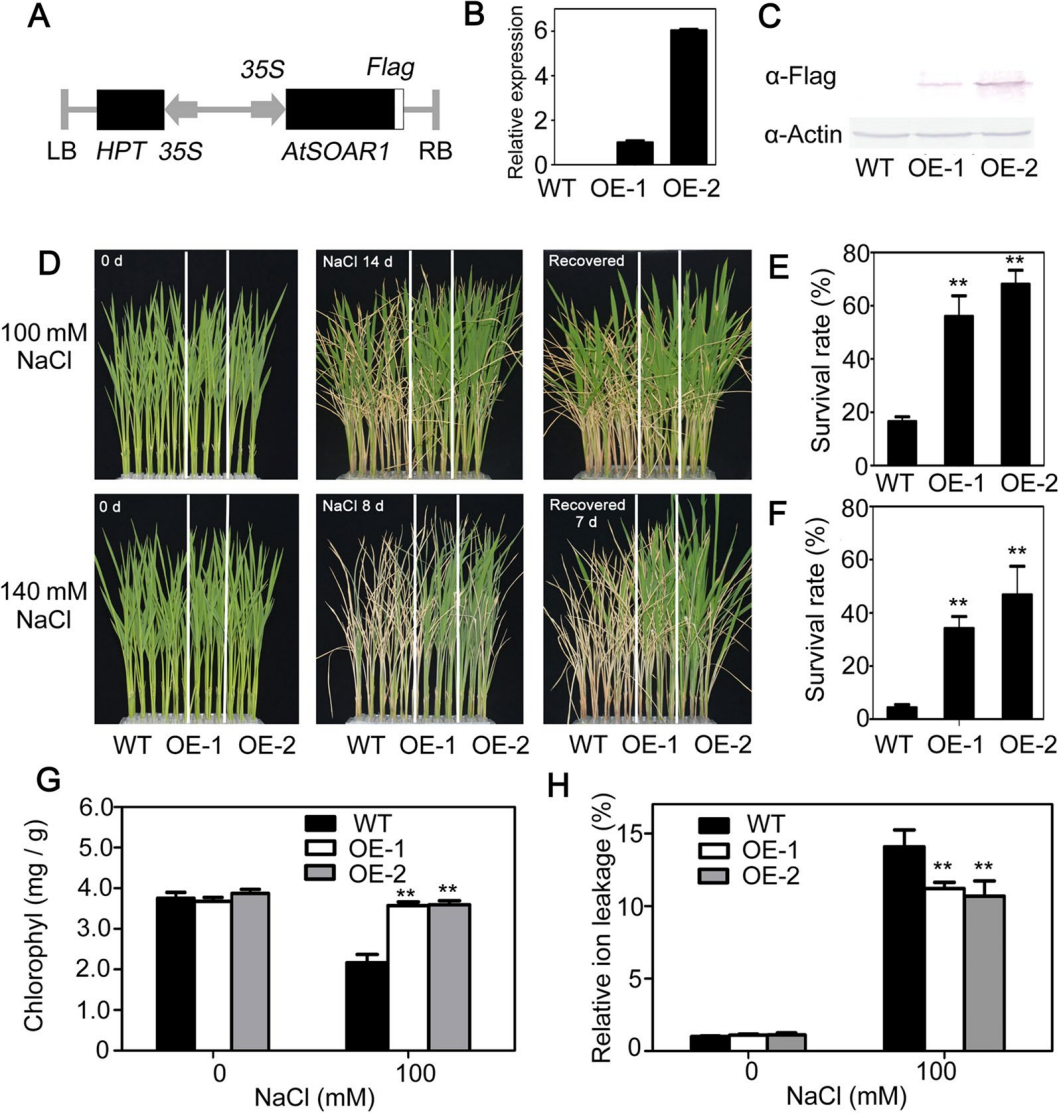
Performance of *AtSOAR1*-transgenic plants under salt stress in early seedling stage. **A** Schematic diagram of the *AtSOAR1*-containing plant expression vector used for rice transformation. **B** Real-time PCR detection of *AtSOAR1* transcripts level in wild-type and the two transgenic lines. Total RNA was extracted from two-week-old seedlings and used for cDNA synthesis. *Actin1* was used for internal control and the expression level of *AtSOAR1* in OE-1 was taken as 1. All the values are means ± SE from three independent biological determinations. **C** Western bolt analysis of AtSOAR1 expression in wild-type and two transgenic lines. Total protein was extracted from two-week-old seedlings and used for immunoblotting. Actin protein was taken as a loading control for immunoblotting. **D** Phenotypic comparison of wild-type and AtSOAR1-transgenic plants OE-1 and OE-2 grown under different concentration of NaCl solution (100 and 140 mM) at the seedling stage. Hydroponic cultured two-week-old seedlings were treated with 100 mM NaCl for 14d or 140 mM NaCl for 8d, and then recovered for 7 d. Three independent biological determinations were conducted and similar results were obtained. **E**, **F** Survival rates of the wild-type and transgenic plants as described in (d) after 7d recovery. All the values are means ± SE from three independent biological determinations. Student’s *t* test was used for comparing the survival rate of each overexpression line with those of wild-type plants (with significant differences at **P < 0.01). **G**, **H** Chlorophyll content **G** and relative ion leakage **H** of the wild-type and transgenic plants under normal and salt stress conditions. Two-week-old seedlings were treated with 100 mM NaCl for 9 d to assay chlorophyll content, or for 24 h to detect relative ion leakage. Student’s *t* test was used for comparing the chlorophyll content or relative ion leakage of each overexpression line with those of wild-type plants (with significant differences at **P < 0.01)

It was reported that overexpression of AtSOAR1 protein in *Arabidopsis* significantly promoted plants tolerance ability to salt in the aspects of seeds germination and seedling growth (Mei et al. 2014; Jiang et al. 2015; Ma et al. 2020). To further investigate and assess the salt-tolerance potential of SOAR1 in rice improvement, we heterologous expressed AtSOAR1 in the local wide-spread *japonica* rice variety NG9108. The full-length coding sequence of *AtSOAR1* was constructed to the 5′ end of the *Flag* tag under the control of the CaMV 35S promoter for producing AtSOAR1-Flag recombinant protein (Fig. 1A). Two *AtSOAR1* transgenic lines, OE-1 and OE-2, were used as representatives for functional analysis in this study, both of which showed high expression level of *AtSOAR1* compared to wild-type as determined by qPCR and western blotting analysis (Fig. 1B, C). The seedlings of wild-type and two *AtSOAR1*-transgenic plants were hydroponically cultured for two weeks which were then treated with 100 mM NaCl for 14 d or 140 mM NaCl for 8 d, respectively. The results showed that no obvious differences were observed between different genotype plants before salt stress, whereas *AtSOAR1*-transgenic plants appeared to be more robust than wild-type plants after salt treatment (Fig. 1D). After recovery for 7d, the number of survived plants of OE-1 and OE-2 was obviously higher than that of wild-type (Fig. 1E, F). Furthermore, we measured the chlorophyll content and relative ion leakage which are two parameters usually used for evaluating the extent of damage to plants under stressful conditions, and observed that *AtSOAR1*-transgenic plants exhibited higher chlorophyll content and lower ion leakage compared with that of wild-type plants under salt stress (Fig. 1G, H). However, no obvious difference in salt-induced inhibition of seed germination was observed between *AtSOAR1*-transgenic plants and wild-type plants (Additional file 1: Fig. S1). These data demonstrated that expression of AtSOAR1 in rice remarkably promoted plant tolerance to salt stress at seedling growth stage.

### Expression of AtSOAR1 in Rice Promotes Grain Productivity Under Salinity Stress

**Fig. 2:**
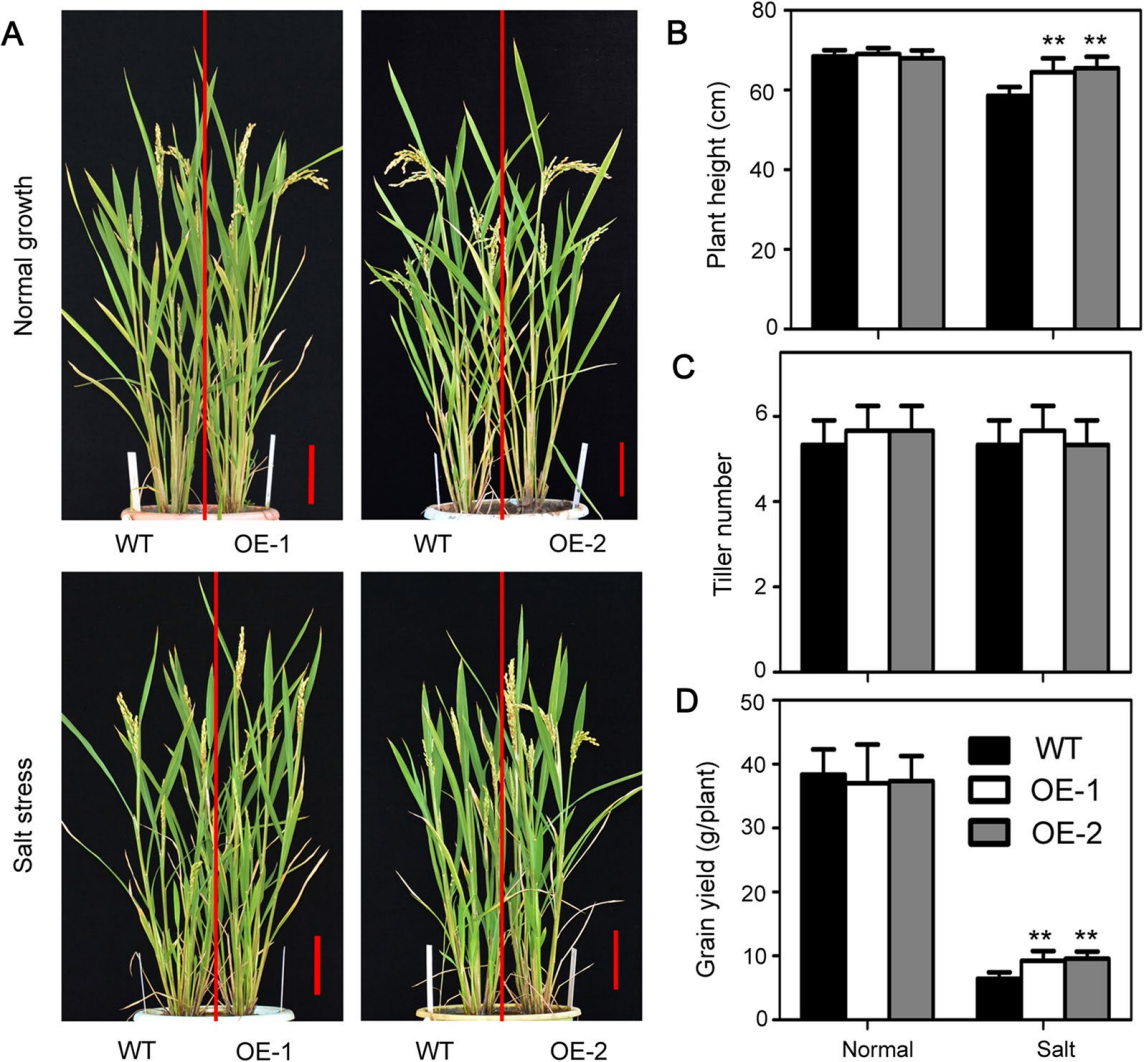
Growth status of the *AtSOAR1*-transgenic plants under salt stress at the reproductive stage. **A** Phenotypic comparison of wild-type and *AtSOAR1*-transgenic plants OE-1 and OE-2 grown under normal and salt stress at the reproductive stage. Two-week-old seedlings were transplanted to pot containing soil with or without 0.15% NaCl (ratio of salt weight to that of dry soil), which were then cultured for total growth period with common agriculture management. Three independent biological determinations were conducted and similar results were obtained. **B**–**D** Statistics of the plant height **B**, tiller number **C** and productivity per plant **D** of wild-type, OE-1 and OE-2 under normal growth and salinity stress conditions. Student’s *t* test was used for comparing the plant height, tiller number or grain yield of each overexpression line with those of wild-type plants (with significant differences at **P < 0.01)

Improving rice yields under salinity stress is one of the main target of salt-tolerance breeding process. To further examine the effects of AtSOAR1 to grain yields under salt tolerance, total growth period experiments for salt tolerance assay were performed. Two-week-old seedlings of wild-type and *AtSOAR1*-transgenic plants were directly planted into soil supplemented with or without NaCl to maximally simulate planting method in salinity soil. Under normal growth conditions, no significant differences were observed between different genotypes plants in the aspects of plant height, tiller numbers and grain yield per plant (Fig. 2A–D). By contrast, when the plants were subjected to salt stress, the plant height and grain yields of OE-1 and OE-2 were higher than that of wild-type plants (Fig. 2A, B, D). Similar tiller numbers between wild-type and transgenic plants were observed under salt stress conditions (Fig. 2C). These results suggest that expression of AtSOAR1 in rice significantly promotes plant growth and yields under salt stress without affecting plant productivity under non-stressful conditions.

### Expression of AtSOAR1 in Rice Results in an ABA-Hypersensitive Phenotype

**Fig. 3:**
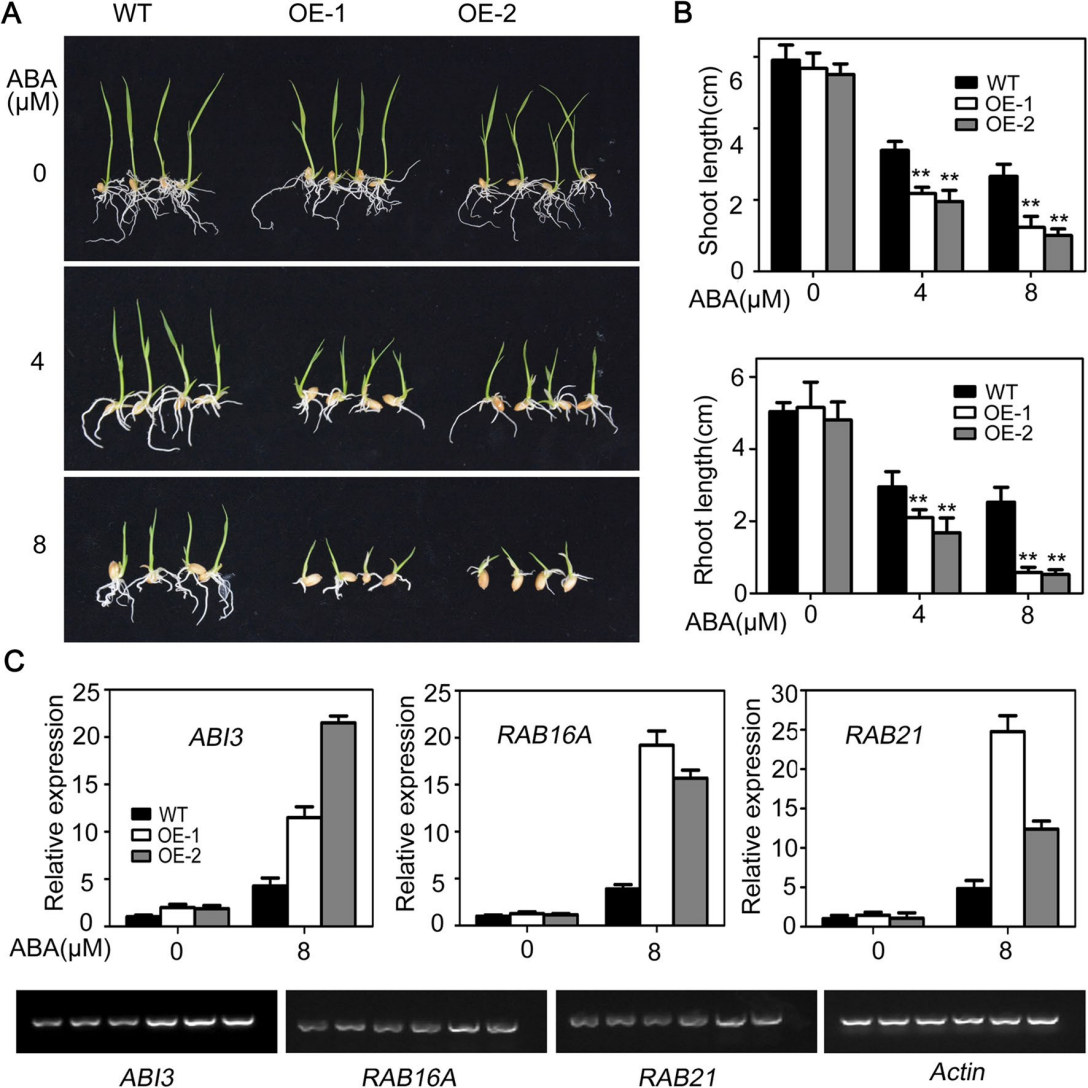
Phenotypes of *AtSOAR1*-transgenic plants in ABA-induced early seedling growth arrest. **A** Phenotypic comparison of wild-type and *AtSOAR1*-transgenic plants OE-1 and OE-2 in ABA-induced inhibition of seedling growth. Germinated seeds of different genotypes were cultured in ABA-free (0 μM) or ( ±) ABA-containing (4 and 8 μM) solutions for 9 d before investigation. The experiments were repeated for three times and similar results were obtained. **B** Quantitative evaluation of shoot and root length of the wild-type and transgenic plants as described in **A**. All the values are means ± SE from three independent biological determinations. Student’s *t* test was used for comparing the shoot length or root length of each overexpression line with those of wild-type plants (with significant differences at **P < 0.01). **C** Detection of ABA-responsive genes in wild-type and *AtSOAR1*-transgenic lines by qPCR and semi-quantitative RT-PCR. The bands from left to right indicates WT without treatment, OE-1 without treatment, OE-2 without treatment, ABA-treated WT, ABA-treated OE-1 and ABA-treated OE-2, respectively. Materials described in **A** was sampled for total RNA extraction and cDNA synthesis. *Actin1* was used for internal control and all the values are means ± SE from three independent biological determinations

The phytohormone ABA plays critical roles in plant response to salt stress and it was reported that overexpression of AtSOAR1 nearly abolished ABA signaling in seed germination and seedling growth (Mei et al. 2014; Jiang et al. 2015; Bi et al. 2019). To investigate whether AtSOAR1 affected ABA signaling in rice, the ABA sensitivity of *AtSOAR1*-transgenic plants were examined and compared with that of wild-type in ABA-induced inhibition of seed germination and seedling growth. We measured ABA sensitivity of wild-type, OE-1 and OE-2 by directly sowing seeds in solutions containing different concentration of ABA, and no significant difference was observed between *AtSOAR1*-transgenic plants and wild-type in seed germination process (Additional file 1: Fig. S1). However, in ABA-mediated seedling growth arrest, both of shoot length and root length were significantly shorter in OE-1 and OE-2 than those of WT plants (Fig. 3A, B). The expression levels of the ABA-responsive genes including *ABI3* (Giraudat et al. 1992), *RAB16A* (Ganguly et al. 2012) and *RAB21* (Lee et al. 2018) were significantly up-regulated in *AtSOAR1*-transgenic plants under ABA treatment, partly explaining the hypersensitive phenotype of AtSOAR1 expression plants (Fig. 3C, D). These results indicated that overexpression of AtSOAR1 in rice results in increased ABA sensitivity in seedling growth, which may contribute to the promoted salt-tolerance ability of the transgenic plants.

### Expression of AtSOAR1 in Rice Alters Global Transcriptomic Profiles

**Fig. 4:**
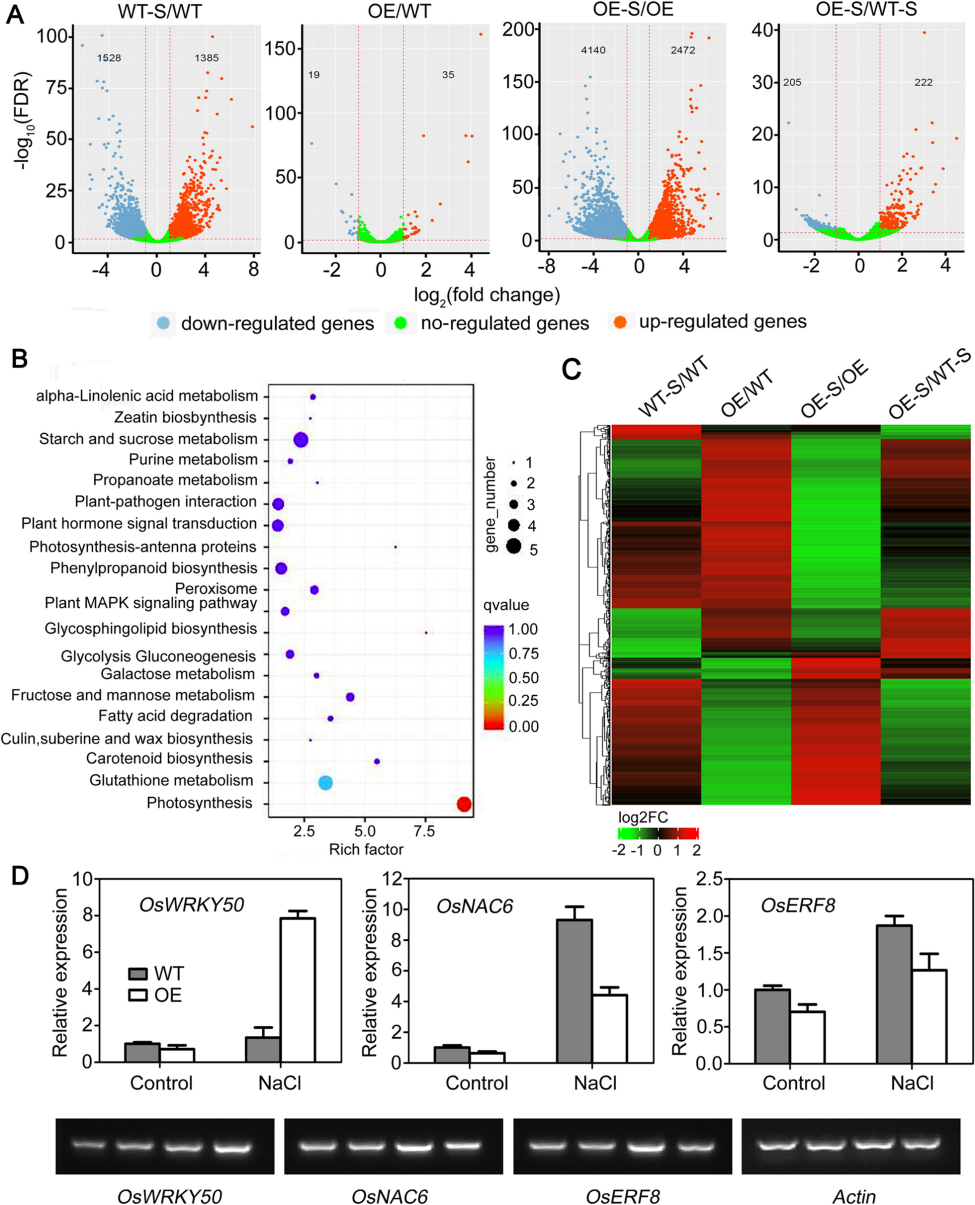
Expression of AtSOAR1 in rice alters global transcriptomic profiles as determined by RNA-seq analysis. **A** Volcano plots showing the status of differential expressed genes between different comparison groups. Red dots represent up-regulated genes and blue dots represent down-regulated genes. Horizontal axis and vertical axis represent the Log2 of fold change and the adjusted *P* value (FDR) of indicated genes, respectively. WT-S/WT, salt-treated WT relative to WT without treatment; OE/WT, OE-1 relative to WT without treatment; OE-S/OE, salt-treated OE-1 relative to OE-1 without treatment; OE-S/WT-S, salt-treated OE-1 relative to salt-treated WT. **B** Statistics of pathway enrichment of differential expressed genes between OE-1 and WT under salt treatment. Each dot represents a KEGG pathway as indicated at the vertical axis. Horizontal axis is rich factor of which value represent significance of enrich level to a certain pathway of differential expressed transcripts. **C** Heat map showing the clustering of the differentially expressed genes of the four comparisons groups. The clustering was drawn based on the normalized, log-scaled FPKM (fragments per kilo base of exon per million reads mapped) values. **D** Validation of the representative differential expressed genes by qPCR and semi-quantitative RT-PCR. The bands from left to right indicates WT without treatment, OE-1 without treatment, salt-treated WT and salt-treated OE-1, respectively. Hydroponic cultured two-week-old seedlings were treated with 140 mM NaCl for 2 d before sampled for total RNA extraction and cDNA synthesis. *Actin1* was used for internal control and all the values are means ± SE from three independent biological determinations

To explore the genes involved in AtSOAR1-mediated salt-stress responsive pathway, we analyzed the transcriptome profiles of wild type and *AtSOAR1*-transgenic plant OE1 with or without NaCl treatment using the Nanopore-based RNA-seq data. Differential expression analysis (fold change ≥ 1.5 and false discovery rate < 0.05) showed that NaCl treatment induced expression change of 2913 genes (1528 down-regulated and 1385 up-regulated) in WT, and 6612 genes (4140 down-regulated and 2472 up-regulated) in OE1 (Fig. 4A; Additional file 1: Fig. S2A; Additional file 2: Data 1). Compared with wild-type plants, expression levels of 54 genes (19 down-regulated and 35 up-regulated) or 427 genes (205 down-regulated and 222 up-regulated) in OE1 were altered under normal growth condition and under NaCl treatment, respectively (Fig. 4A; Additional file 1: Fig. S2A; Additional file 2: Data 1). These results elucidated that expression of SOAR1 in rice leads to change expression of large number of genes in comparison with wild-type plants under salt stress. The differentially expressed genes (DEGs) of the designated four comparison group were used for hierarchical clustering and the results suggested that many of the DEGs showed different response to NaCl between wild-type and *AtSOAR1*-transgenic plant (Fig. 4C). The pathway enrichment was performed to detect whether some DEGs were over-presented on a certain pathway, and the results revealed that salt-induced DEGs in wild-type and SOAR1-induced DEGs under salt stress compared with wild-type were well enriched on multiple pathways, such as photosynthesis, phenylpropanoid biosynthesis, carbon metabolism and phytohormone signal transduction (Fig. 4B; Additional file 1: Fig. S2B). In addition, thirty-three DEGs associated with salt stress were discovered in *SOAR1*-transgenic plant compared with wild-type under NaCl treatment (Additional file 1: Table S2). We confirmed the expression pattern of the three salt-stress responsive genes *OsWRKY50*, *OsNAC6* and *OsERF8* by qPCR, and semi-quantitative RT-PCR, and found that their expression pattern is similar to that of derived from the RNA-seq data (Fig. 4D, E). These results suggest that AtSOAR1 promotes salt tolerance in rice by affecting expression of a series of salt-related genes.

### Expression of AtSOAR1 in Rice Alters Global Alternative Splicing Events

**Fig. 5:**
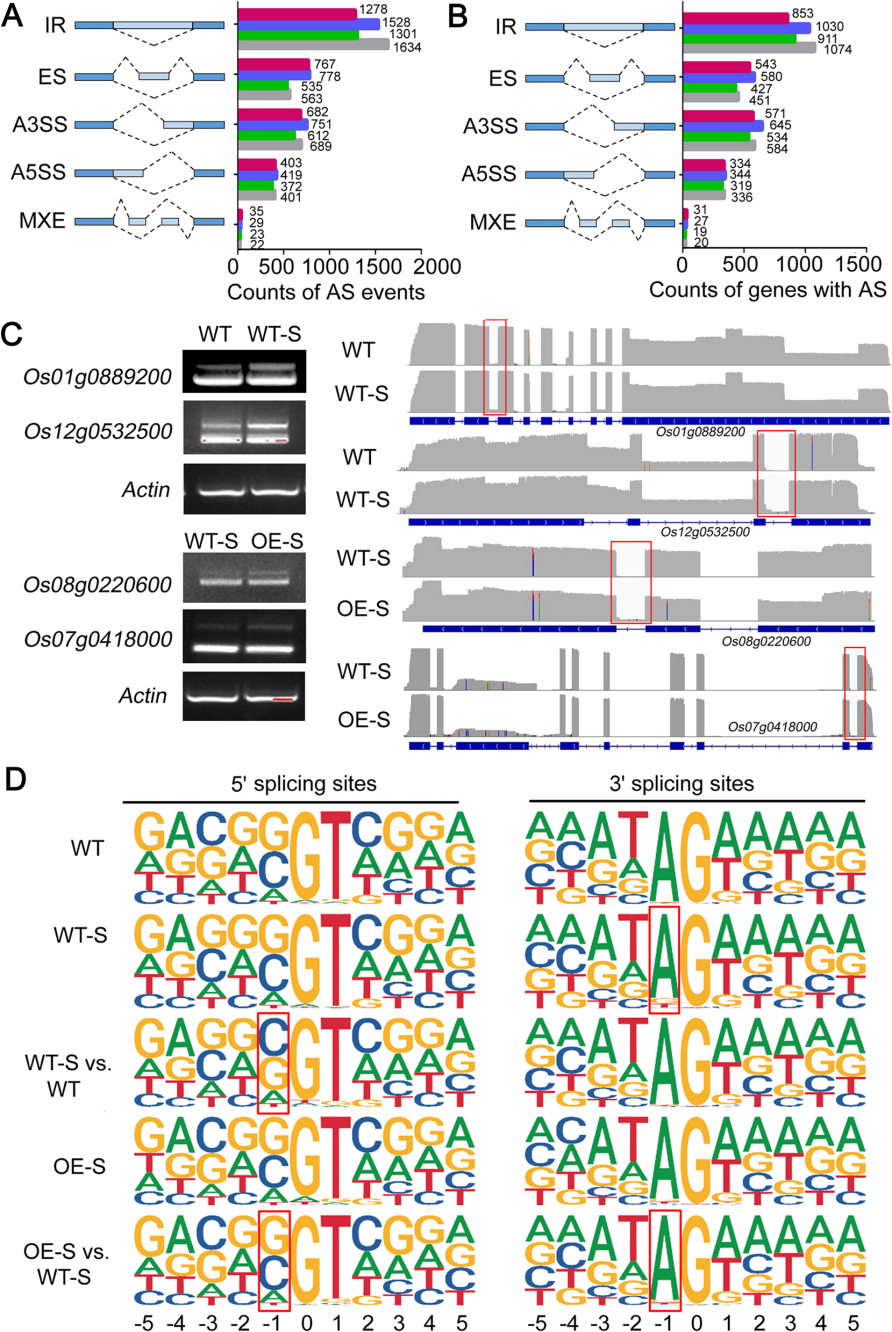
Global alternative splicing analysis of wild-type and *AtSOAR1*-transgenic plants under salt stress. **A** Statistics of different kinds of alternative splicing (AS) events in wild-type and OE-1 under normal and salt stress conditions. **B** Statistics of genes corresponding to different kinds of AS events. The red, blue, green and grey bars indicate salt-treated OE-1, salt-treated WT, OE-1 without treatment and WT without treatment, respectively, in (**A**) and (**B**). **C** Representative differential alternative splicing between WT and WT-S, or WT-S and OE-S were validated by RT–PCR and showed by IGV browser. The retained intron regions were marked by red box. Hydroponic cultured two-week-old seedlings were treated with 0 or 140 mM NaCl for 2 d followed by RNA extraction and cDNA synthesis. WT, wild-type plants without NaCl treatment; WT-S, wild-type plants with NaCl treatment; OE, *AtSOAR1*-transgenic plants OE-1 without NaCl treatment; OE-S, *AtSOAR1*-transgenic plants OE-1 with NaCl treatment. *Actin 1* was taken as control for RT-PCR. **D** Frequency distribution of nucleotides around the splicing sites. The nucleotide sequences represent the consensus sequences of 5′- and 3′- splicing sites and the size of the letter indicates frequency of certain nucleotide. WT, WT-S and OE-S means nucleotides distribution around the splicing sites in wild-type, salt-treated wild-type and salt-treated OE-1 plants. WT-S/WT means nucleotides distribution around the splicing sites of salt-induced splicing events in wild-type. OE-S/WT-S means nucleotides distribution around the splicing sites of the new salt-induced splicing events in OE-1 compared with that of wild-type plants. Red boxes marked the altered frequency of the dominant A at position − 1 of 3′ splicing sites or the frequency of the C and G at position − 1 of the 5′ splicing sites

It was reported that the RNA-binding protein AtSOAR1 was involved in regulating alternative splicing of a subset of genes involved in stress signaling (Bi et al. 2019; Ma et al. 2020). To determine whether AtSOAR1 expression in rice induce pre-mRNA splicing alteration, we performed Nanopore-based full-length transcriptome analysis. The reads number of different samples ranked from 6.3 to 9.9 million with mean length 882 to 1135 bp and all of the full-length reads percentage were more than 88.21 (Additional file 1: Table S1). By comparing the alternative splicing (AS) events detected in this study, including intron retention (IR), exon skipping (ES), alternative 3′ splice site (A3SS), alternative 5′ splice site (A5SS) and mutually exclusive exon (MXE), we found that the main types of AS events involved IR, and that the number of IR and its corresponding genes in *SOAR1*-transgenic plants were less than that in wild-type plants under salt and normal growth conditions, whereas salt stress increased the number of ES events and its corresponding genes both in wild-type and SOAR1 expression plants (Fig. 5A, B). Besides, the results showed that salt stress induced 2397 AS events containing 2168 IR, and that SOAR1 expression induced 1934 AS events containing 1893 IR under salt stress conditions, suggesting that both salt stress and SOAR1 triggered global alternative splicing with overwhelming majority of events involved IR (Additional file 1: Fig. S3; Additional file 3: Data 2). Representative differential alternative splicing events were validated by RT–PCR using primers flanking the splice sites (Fig. 5C). Furthermore, we detected the consensus sequences at 5′ and 3′ splicing sites which is critical for pre-mRNAs splicing and found that the frequency of the dominant A at position − 1 of 3′ splicing sites in salt-treated wild-type plants was less that of wild-type plants without treatment, and that the frequency of the C and G at position − 1 of the 5′ splicing sites in the novel splicing events (WT-S vs. WT) differed from the consensus sequence, suggesting that salt stress induce abnormal splicing of pre-mRNA. However, the dominant A at position − 1 of 3′ splicing sites and the C and G at position − 1 of the 5′ splicing sites in the salt-treated AtSOAR1 overexpression plants and novel splicing events (OE-S vs. WT-S) recovered to the conserved sequence (Fig. 5D). These results imply that AtSOAR1 expression decreased the adverse effect of salt stress on pre-mRNA splicing, which is consistent with the positive role of SOAR1 in promoting plants salt-tolerance ability.

### Expression of OsSOAR1 (The Homologous Gene of SOAR1 in Rice) Enhances Salt Tolerance at Seedling Growth Stage

**Fig. 6:**
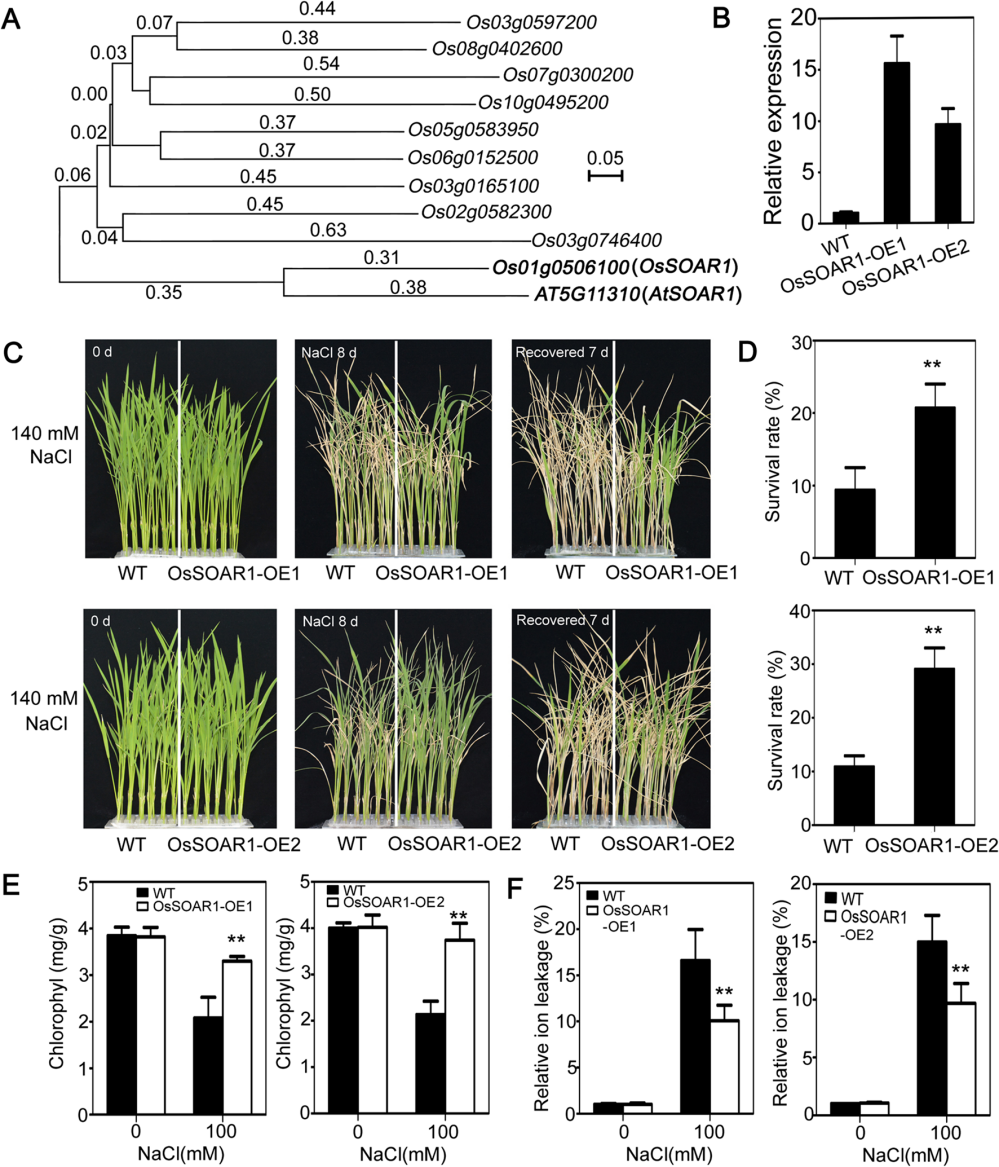
Performance of *OsSOAR1*-transgenic plants under salt stress at early seedling stage. **A** Phylogenic analysis of *AtSOAR1* with the homozygous *PPR* genes in rice using the neighbor-joining method with MEGA version 11 by alignment of the gene sequences with ClustalW. **B** Real-time PCR detection of *OsSOAR1* transcripts level in wild-type and the two transgenic lines OsSOAR1-OE1 and OsSOAR1-OE2. Total RNA was extracted from two-week-old seedlings and used for cDNA synthesis. *Actin1* was used for internal control and the expression level of *OsSOAR1* in WT was taken as 1. All the values are means ± SE from three independent biological determinations. **C** Phenotypic comparison of wild-type and *OsSOAR1*-transgenic plants OsSOAR1-OE1 and OsSOAR1-OE2 grown under 140 mM NaCl solution at the seedling stage. Hydroponic cultured two-week-old seedlings were treated with 140 mM NaCl for 8d, and then recovered for 7d. Three independent biological determinations were conducted and similar results were obtained. **D** Survival rates of the wild-type and transgenic plants as described in **C** after 7d recovery. All the values are means ± SE from three independent biological determinations. Student’s *t* test was used for comparing the survival rate of each overexpression line with those of wild-type plants (with significant differences at **P < 0.01). **E**, **F** Chlorophyll content **E** and relative ion leakage **F** of the wild-type and transgenic plants under normal and salt stress conditions. Two-week-old seedlings were treated with 100 mM NaCl for 9 d to assay chlorophyll content, or for 24 h to detect relative ion leakage. Student’s *t* test was used for comparing the chlorophyll content or relative ion leakage of each overexpression line with those of wild-type plants (with significant differences at **P < 0.01)

To explore whether the homologous gene of *AtSOAR1* in rice function in controlling plants salt tolerance, we analyzed the phylogenic relationship of *AtSOAR1* with the homologous *PPR* genes in rice. The comparison of *AtSOAR1* gene sequences with those of rice genes showed that *Os01g0506100*, designated as *OsSOAR1*, shared the highest identity with *AtSOAR1* (Fig. 6A; Additional file 1: Fig. S4). Then, we overexpressed *OsSOAR1* in rice and observed that the two representative transgenic lines (OsSOAR1-OE1 and OsSOAR1-OE2) displayed salt-tolerance phenotype at seedling growth stage (Fig. 6B–D). Measurement of physiological indicators showed that *OsSOAR1* transgenic plants exhibited higher chlorophyll content and lower ion leakage compared with that of wild-type plants under salt stress (Fig. 6E, F). These data suggest that OsSOAR1 is positively involved in plants response to salt stress in rice.

### Identification of Salt-and Other Abiotic Stress-Induced SOAR1-like PPR Genes in Rice

**Fig. 7:**
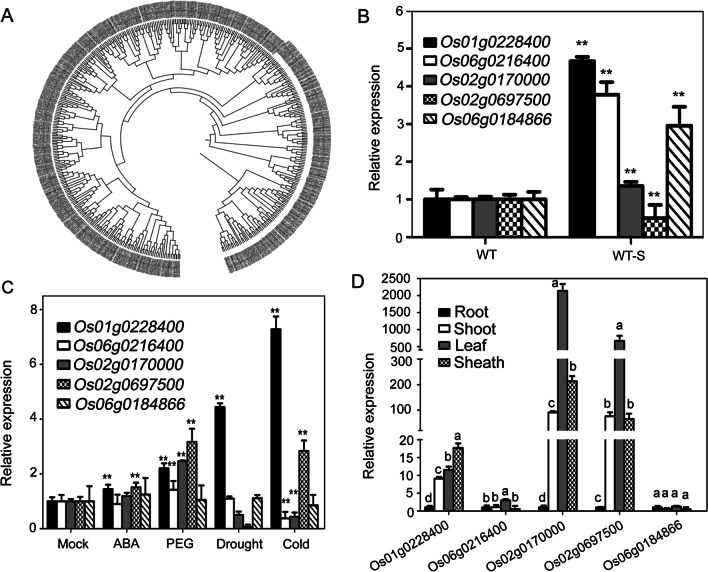
Expression pattern of the five salt-induced *PPR* genes. **A** Phylogenic analysis of the rice *PPR* genes identified in this study using the neighbor-joining method with MEGA version 11 by alignment of the gene sequences with MUSCLE algorithm. **B** Confirmation of the salt-induced *PPR* genes of RNA-seq data by qPCR assays. Hydroponic cultured two-week-old seedlings were treated with 0 or 140 mM NaCl for 2d followed by RNA extraction and cDNA synthesis. Expression level of each gene without salt stress was taken as 1. *Actin1* was used for internal control and all the values are means ± SE from three independent biological determinations. Student’s *t* test was used for comparing the relative expression of each *PPR* genes of wild-type plants under salt stress with those of wild-type plants grown under non-stressful conditions. **C** Expression of the five salt-induced *PPR* genes in response to ABA, PEG, drought and cold treatment. Hydroponic cultured two-week-old seedlings were treated with 0 or 10 µM ( ±) ABA, 100 mM PEG and cold stress for 5 h or drought stress for 2 h. The material was collected at the indicated time and used for total RNA extraction. Expression level of each gene without stress treatment was taken as 1. *Actin1* was used for internal control and all the values are means ± SE from three independent biological determinations. Student’s *t* test was used for comparing the relative expression of each *PPR* genes of wild-type plants under stress treatments with those of wild-type plants grown under non-stressful conditions. **D** Expression level the five *PPR* genes were detected in different tissues by qPCR assays. Expression level of each gene in root was taken as 1. *Actin1* was used for internal control and all the values are means ± SE from three independent biological determinations. Duncan’s multiple range test was used and different letters represent significant differences at *P* < 0.05

To identify the salt responsive SOAR1-like *PPR* genes, we first searched all of the *PPR* genes according to our RNA-seq data. The results showed that 446 *PPR* genes were detected in our study and five *PPRs* transcripts were induced or inhibited by NaCl treatment, which were further confirmed by qPCR and semi-quantitative RT-PCR (Fig. 7A, B; Additional file 1: Fig. S5A). We detected the five *PPRs* expression pattern in response to ABA, PEG, drought and cold treatments to analysis their physiological functions. Our results showed that the *PPR* genes, *Os01g0228400*, was induced by salt and other abiotic stress, while the transcripts of *Os06g0216400* and *Os02g0170000* was induced by salt, PEG and drought, but inhibited by cold stress (Fig. 7B, C; Additional file 1: Fig. S5B). The expression of *Os02g0697500* was inhibited by salt stress specially which were induced by all of other treatment, and the expression of *Os06g0184866* was affected by salt only (Fig. 7B, C; Additional file 1: Fig. S5B). We analyzed the tissue expression of the five *PPR* genes and found that the expression of *Os01g0228400*, *Os02g0170000* and *Os02g0697500* differed greatly, which were mainly expressed in aerial parts (Fig. 7D; Additional file 1: Fig. S5C). These results indicated that the newly identified *PPR* genes may involve in plants response to salt or other abiotic stress.

## Discussion

### Both Heterologous Expression of AtSOAR1 and Homologous Expression of OsSOAR1 Confers Salt Tolerance in Rice

It was reported that the *AtSOAR1*-overexpression lines in *Arabidopsis* showed strong tolerance phenotype to salt, drought and cold stresses, especially with surprisingly resistance to high concentration of NaCl similar to that of seawater in germination and post-germination growth (Mei et al. 2014; Jiang et al. 2015). In this study, we heterologous expressed AtSOAR1 in the local wide-spread *japonica* rice variety NG9108 to check its application value in crop improvement under salinity conditions and observed that the transgenic plants overexpression AtSOAR1 exhibited salt tolerance phenotype at seedling growth stage as indicated by the higher survival rate, higher chlorophyll content and lower relative ion leakage of the transgenic plants compared with that of wild-type under salt stress (Fig. 1). Total growth period experiments for salt tolerance assay indicated that expression of AtSOAR1 did not affect the agricultural traits, such as plant height, tiller numbers and grain yield per plant under non-stressful conditions, but promoted plant height and grain yield per plant under salt stress, implying that AtSOAR1 confers salt tolerance in rice and *Arabidopsis* and was likely to be used for artificial improvement of crops (Fig. 2A, B, D). Though highly resistant phenotype to salt stress was observed in *AtSOAR1*-transgenic *Arabidopsis* plants in seed germination stage, no obvious difference was observed in *AtSOAR1*-transgenic rice in the salt-induced inhibition of seed germination process, suggesting that AtSOAR1-mediated mechanism to salt in germination stage was different between monocotyledons and dicotyledons plants (Additional file 1: Fig. S1). To explore whether the homologous gene of *SOAR1* exits in rice and whether it play roles in plants resistance to salt stress, we aligned in *japonica* rice genome with the sequence of *AtSOAR1* and found out the most closely related gene of *SOAR1*, *Os01g0506100*, designated as *OsSOAR1* (Fig. 6A; Additional file 1: Fig. S4). Transgenic lines overexpression *OsSOAR1* displayed the salt tolerance phenotype at seedling growth stage and exhibited higher chlorophyll content and lower ion leakage compared with that of wild-type plants under salt stress, suggesting that OsSOAR1 also plays positive roles in response to salt stress. 

### How does SOAR1 Function in Response to Salt Stress in Rice?

**Fig. 8:**
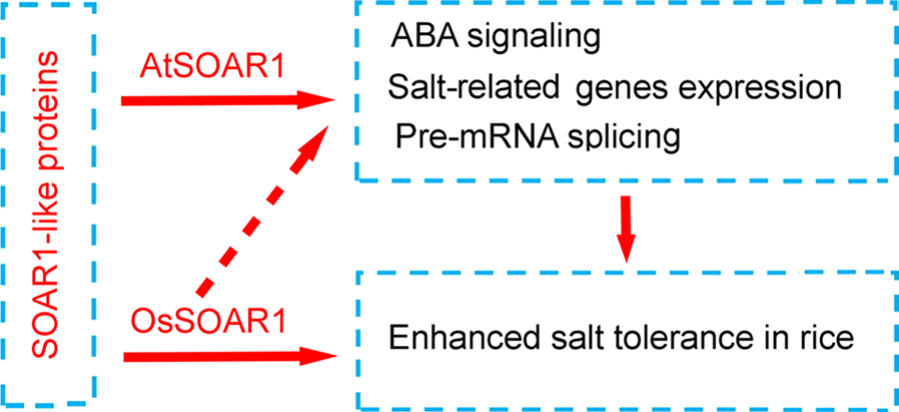
A schematic diagram of SOAR1-like proteins function under salt stress. SOAR1-like proteins positively modulate plants response to salt stress through influencing ABA signaling, salt-responsive genes expression and pre-mRNA splicing. Solid lines indicate confirmed results and dotted lines indicate unconfirmed results

It is well known that salt stress induces ABA accumulation and ABA signaling to modulate stress-related gene expression which contribute to plants adaptation to salt stress (Zhu et al. 2002; Van et al. 2020). Previous studies demonstrated that SOAR1 is a negative regulator in ABA signaling and the AtSOAR1-overexpression *Arabidopsis* plants showed dramatically insensitive phenotype to ABA. However, the transgenic rice exhibited increased ABA sensitivity in ABA-induced inhibition of seedling growth, but no obvious difference was observed in ABA-induced inhibition of seed germination, which partially explained the salt-tolerance phenotype of AtSOAR1 overexpression plants (Fig. 3; Additional file 1: Fig. S1). We conducted full-length transcriptome analysis to explore the molecular mechanisms of promoted salt-tolerance ability of AtSOAR1 overexpression plants and found that expression of AtSOAR1 in rice induced global transcriptomic profiles alteration including a subset of salt-responsive genes (Fig. 4A, C; Additional file 1: Fig. S2A; Additional file 1: Table S2). Totally 427 differentially expressed genes (205 down-regulated and 222 up-regulated) between AtSOAR1 overexpression line and wild-type plants under salt stress were enriched to multiple pathways, such as photosynthesis, phenylpropanoid biosynthesis, carbon metabolism and phytohormone signal transduction, which may contribute to resistance to salt stress through a couple of different mechanisms (Fig. 4B; Additional file 1: Fig. S2B; Additional file 2: Data 1).Pre-mRNA splicing is one of key post-transcriptional regulatory mechanism in regulating gene expression and plant response to abiotic stresses (Ling et al. 2017). It was reported that AtSOAR1 binds the mRNA of *ABA INSENSITIVE5* (*ABI5*) to repress translation of ABI5 and cooperates with U6 biogenesis protein 1 (USB1) to regulate ABA signaling by affecting spliceosome assembly (Bi et al. 2019; Ma et al. 2020). The results of full-length transcriptome analysis revealed that AtSOAR1 expression in rice triggered global alternative splicing with overwhelming majority of events involved IR under salt stress, and AtSOAR1 expression decreased the number of AS events and its corresponding genes both under salt and normal growth conditions (Fig. 5; Additional file 1: Fig. S3). Interestingly, the stress-associated *RAB21* gene which has been reported to be alternative spliced under salt stress, was differentially alternative spliced between *AtSOAR1-*overexpression plants and wild-type under salt stress, implying that AtSOAR1 influences the alternative splicing of *RAB21* (Li et al. 2018). However, the differential alternative splicing events and genes between AtSOAR1 overexpression plants and wild-type under salt stress was analyzed but the *OsABI5* gene was not included, suggesting that the target pre-mRNA and function mechanism of AtSOAR1 in rice and *Arabidopsis* was different (Fig. 5A–C; Additional file 3: Data 2). Moreover, AtSOAR1 expression in rice decreased the abnormal splicing of pre-mRNA under salt stress at the dominant A at position − 1 of 3′ splicing sites and the C and G at position − 1 of the 5′ splicing sites, which may contribute to the enhanced salt tolerance ability of AtSOAR1-transgenic rice (Fig. 5D). Taken together, all these findings suggested that the SOAR1-like proteins were positively involved in plants response to salt stress in rice through influencing ABA signaling, salt-responsive genes expression and pre-mRNA splicing under salt stress though the function mechanisms of OsSOAR1 still need to be illustrated, implying that it possesses potential utilization value in improving the salt-tolerance trait of crops (Fig. 8). However, SOAR1-mediated molecular mechanisms upon salt stress in rice should be further studied, which will help to discover novel salt-related genes and understand the highly complicated salt stress signaling network. Given that the homologous gene of SOAR1 in rice, *OsSOAR1*, plays positive regulatory roles in response to salt stress, whether the working model of AtSOAR1 in *Arabidopsis* was applied to that of OsSOAR1 in rice need to be explored to fully understand the biology function of SOAR1. Expression level of *OsSOAR1* was not altered upon salt and other stress treatments, implying that the function of *OsSOAR1* was not regulated through transcriptional level and may be activated through the process of post-translational modification under stress conditions (Additional file 1: Fig. S6). Besides, whether the newly discovered salt-induced *PPR* genes function in plants resistance to salt stress still need to be elucidated further.

## Conclusion

In summary, our results demonstrated that the SOAR1-like proteins, including the *Arabidopsis* SOAR1 (AtSOAR1) and its homologous protein in rice (OsSOAR1), are positively involved in plant response to salt stress and may be used for crop improvement in rice under salinity conditions through transgenic manipulation.

## Supplementary Information

**Additional file 1**. **Fig. S1**: Phenotypic comparison of wild-type and SOAR1-transgenic plants OE-1 and OE-2 in NaCl- or ABA- induced inhibition of seed germination. **Fig. S2**: Statistics and analysis of differential expressed genes between different comparison groups. **Fig. S3**: Salt-induced and SOAR1-mediated alternative splicing analysis. **Fig. S4**: Alignment of coding sequence of AtSOAR1 and OsSOAR1. **Fig. S5**: Confirmation of the expression pattern of the five salt-induced PPR genes by semi-quantitative RT-PCR. **Fig. S6**: Expression pattern analysis of OsSOAR1 under abiotic stress treatments by qPCR. **Table S1**: Summary of the RNA-Seq data. **Table S2**: List of differential expressed (≥ 2 fold) salt-responsive genes in SOAR1 expression plants compared with that of wild-type plants under NaCl treatment by GO analysis. **Table S3**: PCR primers used in this study.

**Additional file 2**. **Data 1**: List of differential expressed (≥ 2 fold) genes in SOAR1 expression plants compared with that of wild-type plants with or without NaCl treatment.

**Additional file 3**. **Data 2**: List of salt stress and SOAR1 triggered global alternative splicing events.

## Data Availability

All sequencing raw data have been deposited to NCBI with a ID number of PRJNA869885 and other data supporting this study are available from the corresponding authors based on reasonable request.

## Abbreviations

PPR: Pentatricopeptide repeat proteins
SOAR1: Suppressor of the ABAR-overexpressor 1
ABA: Abscisic acid
AS: Alternative splicing
IR: Intron retention
ES: Exon skipping
A3SS: Alternative 3′ splice site
A5SS: Alternative 5′ splice site
MXE: Mutually exclusive exon
