# Supplementary material for: The PPR-Domain Protein SOAR1 Regulates Salt Tolerance in Rice
Source: Rice (N Y). 2022 Dec 3;15:62. doi: 10.1186/s12284-022-00608-x (PMC9719575; doi:10.1186/s12284-022-00608-x)
Supplement: Supplementary file 1 — Additional file 1. Fig. S1: Phenotypic comparison of wild-type and SOAR1-transgenic plants OE-1 and OE-2 in NaCl- or ABA- induced inhibition of seed germination. Fig. S2: Statistics and analysis of differential expressed genes between different comparison groups. Fig. S3: Salt-induced and SOAR1-mediated alternative splicing analysis. Fig. S4: Alignment of coding sequence of AtSOAR1 and OsSOAR1. Fig. S5: Confirmation of the expression pattern of the five salt-induced PPR genes by semi-quantitative RT-PCR. Fig. S6: Expression pattern analysis of OsSOAR1 under abiotic stress treatments by qPCR. Table S1: Summary of the RNA-Seq data. Table S2: List of differential expressed (≥ 2 fold) salt-responsive genes in SOAR1 expression plants compared with that of wild-type plants under NaCl treatment by GO analysis. Table S3: PCR primers used in this study. [file 12284_2022_608_MOESM1_ESM.docx]

**Supplementary Data**

Lu et al., The PPR-domain protein SOAR1 regulates salt tolerance with potential value in crop improvement for salt tolerance in rice


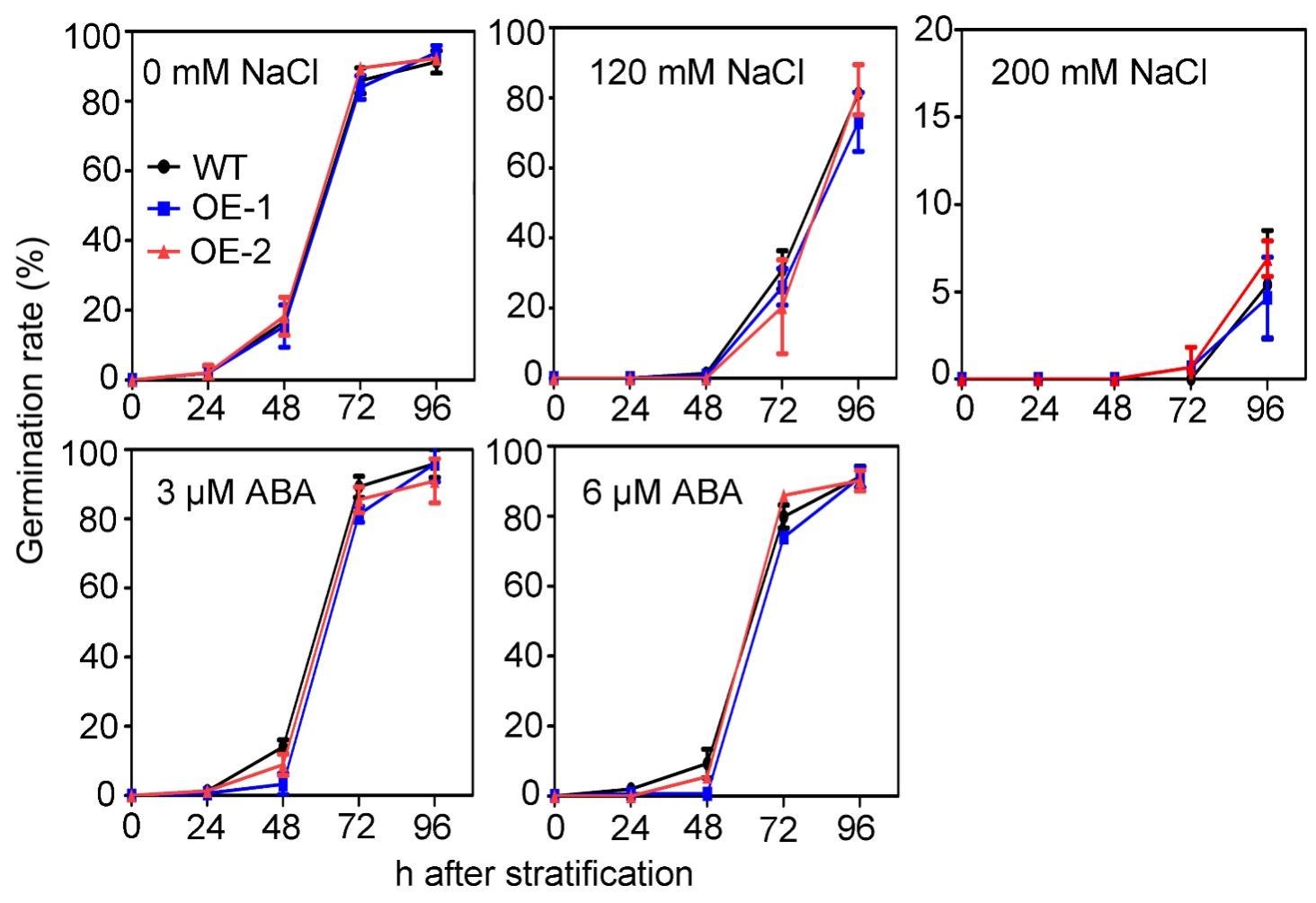


**Supplementary Figure. S1.** Phenotypic comparison of wild-type and *AtSOAR1*-transgenic plants OE-1 and OE-2 in NaCl- or ABA- induced inhibition of seed germination. Seeds of different genotypes were cultured in solutions supplemented with different concentrations of NaCl (0, 120, 200 mM NaCl) or (±) ABA (3, 6 μM). Numbers of germinated seeds were scored from 0-96 h and germination rates were calculated. All the values are means ± SE from three independent experiments.


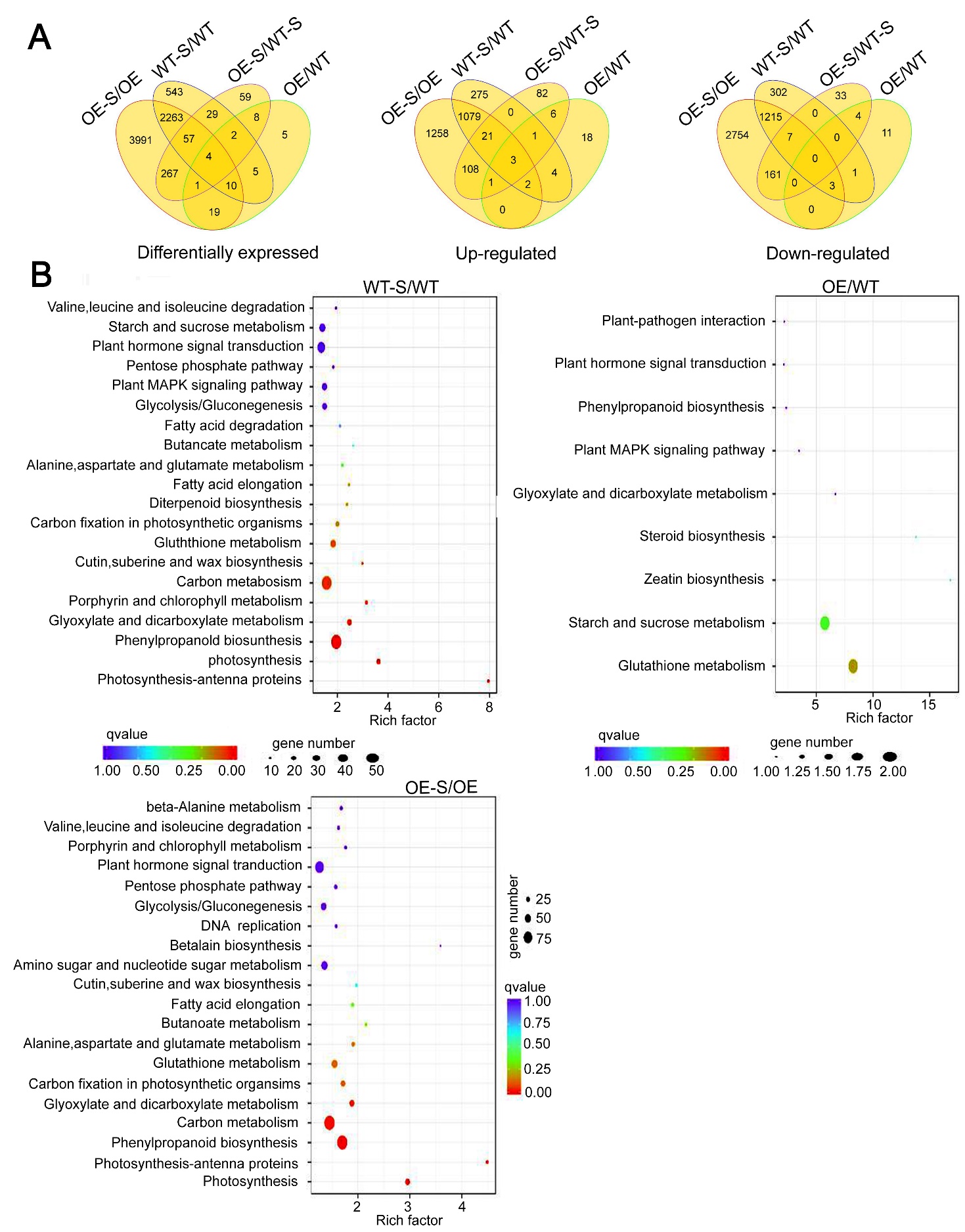


**Supplementary Figure. S2.** Statistics and analysis of differential expressed genes between different comparison groups. (A) Venn diagram showing the number of differential expressed-, up-regulated-, and down-regulated-genes, and the overlaps of genes between different comparison groups. (B) Statistics of pathway enrichment of differential expressed genes between different comparison groups. Upper left panel, salt-treated WT relative to WT without treatment; upper right panel, OE-1 relative to WT without treatment; lower left panel, salt-treated OE-1 relative to OE-1 without treatment.

**
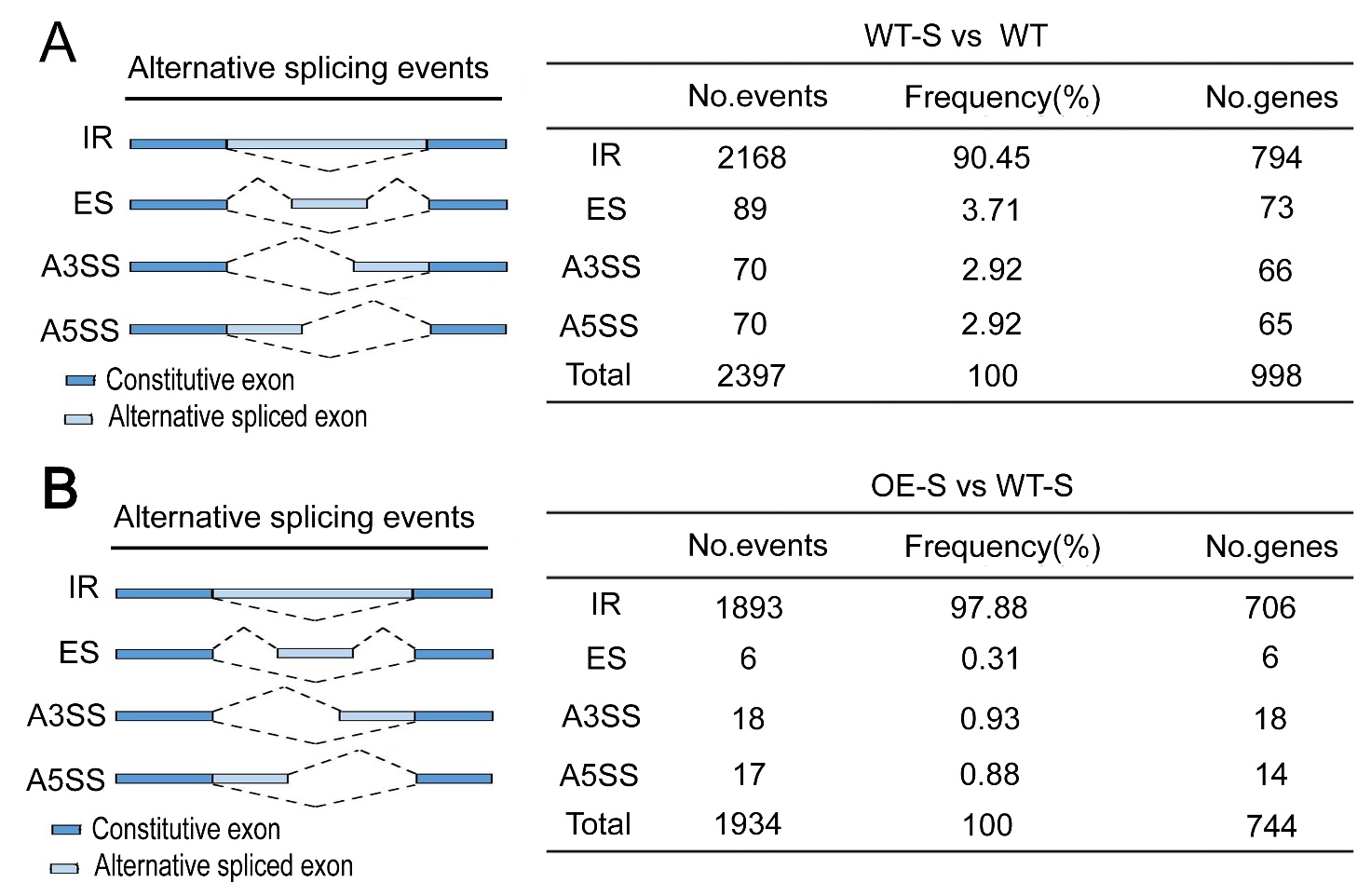
**

**Supplementary Figure. S3.** Salt-induced and AtSOAR1-mediated alternative splicing analysis. (A) Global analysis of salt-induced alternative splicing. WT, wild-type plants without NaCl treatment; WT-S, wild-type plants with NaCl treatment; OE, SOAR1-transgenic plants OE-1 without NaCl treatment; OE-S, *AtSOAR1*-transgenic plants OE-1 with NaCl treatment. (B) Global analysis of AtSOAR1-mediated alternative splicing.


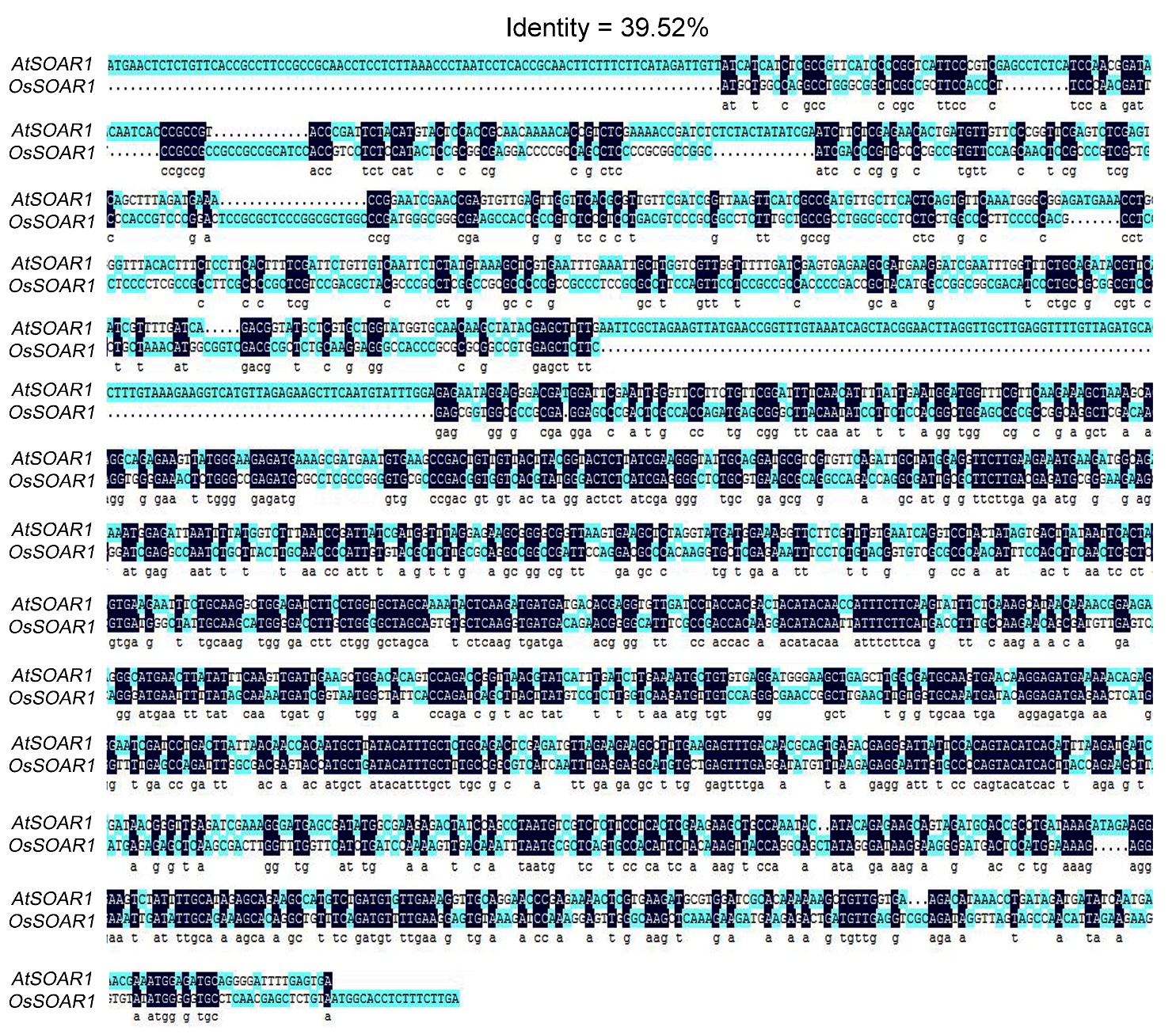


**Supplementary Figure. S4.** Alignment of coding sequence of *AtSOAR1* and *OsSOAR1*. The sequences were compared using software DNAMAN4.0 version. The conserved sequences were marked by black color.


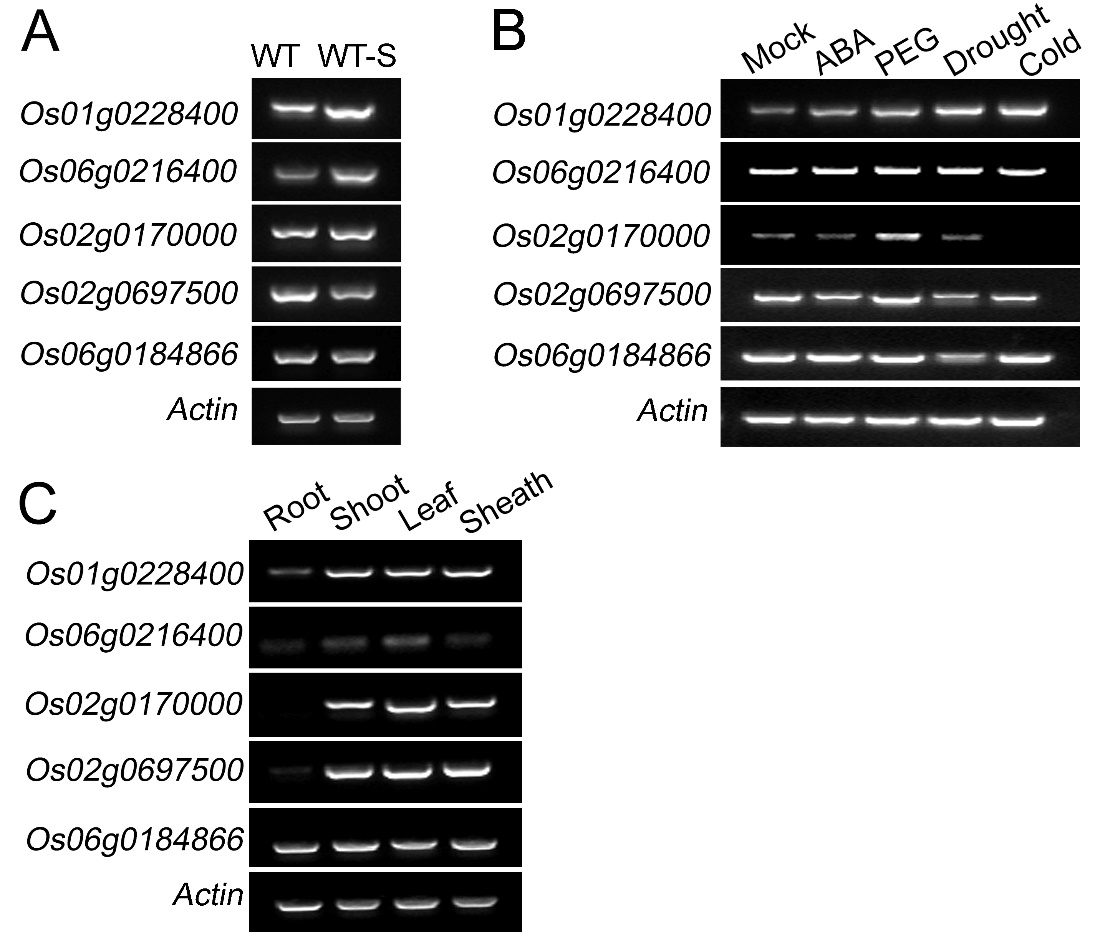


**Supplementary Figure. S5.** Confirmation of the expression pattern of the five salt-induced *PPR* genes by semi-quantitative RT-PCR. (A) Detection of the salt-induced *PPR* genes by semi-quantitative RT-PCR. Hydroponic cultured two-week-old seedlings were treated with 0 or 140 mM NaCl for 2d followed by RNA extraction and cDNA synthesis. WT-S, salt-treated wild-type. *Actin1* was used for internal control. The experiments were repeated for three times and similar results were obtained. (B) Detection of the five salt-induced *PPR* genes in response to ABA, PEG, drought and cold treatment. Hydroponic cultured two-week-old seedlings were treated with 0 or 10 µM (±) ABA, 100 mM PEG and cold stress for 5 h or drought stress for 2 h. The material was collected at the indicated time and used for cDNA synthesis. *Actin1* was used for internal control. (C) Detection of the expression level of the five *PPR* genes in different tissues by semi-quantitative RT-PCR. *Actin1* was used for internal control.


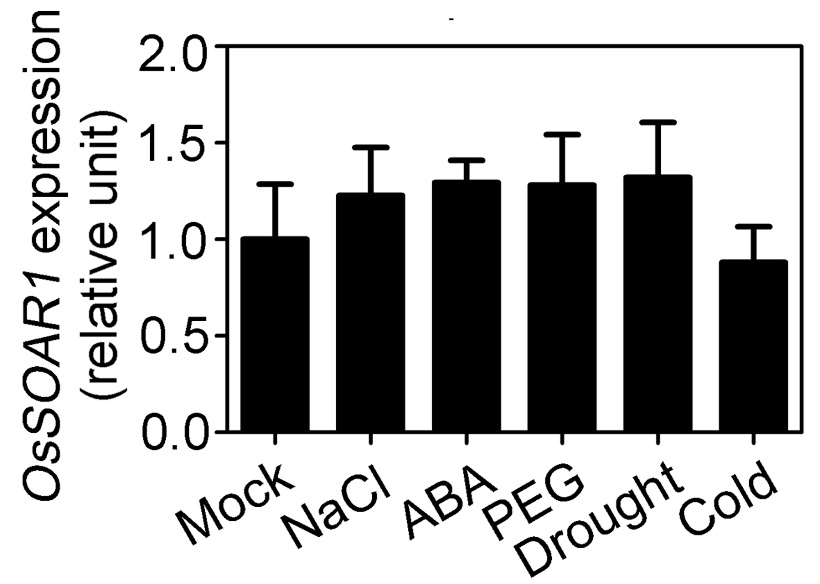


**Supplementary Figure. S6.** Expression pattern analysis of *OsSOAR1* under abiotic stress treatments by qPCR. Hydroponic cultured two-week-old seedlings were treated with 0 or 10 µM (±) ABA, 100 mM PEG and cold stress for 5 h or drought stress for 2 h. The material was collected at the indicated time and used for total RNA extraction. Expression level of *OsSOAR1* without stress treatment was taken as 1. *Actin1* was used for internal control and all the values are means ± SE from three independent biological determinations.

**Supplementary Table S1.** Summary of the RNA-Seq data.

|  | **Reads** | **Mean Length** | **Clean Reads** | **Full-length Reads** | **Full-length Percentage (%)** |
| --- | --- | --- | --- | --- | --- |
| **WT-Rep.1** | 6,339,743 | 1,080 | 6,137,755 | 5,420,529 | 88.31 |
| **WT-Rep.2** | 6,742,849 | 1,038 | 6,513,108 | 5,764,424 | 88.50 |
| **WT-Rep.3** | 7,623,044 | 1,012 | 7,379,743 | 6,550,015 | 88.76 |
| **OE-Rep.1** | 7,662,826 | 945 | 7,430,128 | 6,627,106 | 89.19 |
| **OE-Rep.2** | 6,878,140 | 1,020 | 6,645,954 | 5,920,838 | 89.09 |
| **OE-Rep.3** | 7,376,458 | 1,019 | 7,152,951 | 6,377,889 | 89.16 |
| **WT-S-Rep.1** | 7,100,127 | 1,135 | 6,847,572 | 6,074,821 | 88.71 |
| **WT-S-Rep.2** | 7,450,057 | 1,014 | 7,202,940 | 6,422,619 | 89.17 |
| **WT-S-Rep.3** | 7,356,668 | 924 | 7,115,010 | 6,391,654 | 89.83 |
| **OE-S-Rep.1** | 7,377,367 | 941 | 7,131,639 | 6,456,436 | 90.53 |
| **OE-S-Rep.2** | 9,430,535 | 933 | 9,118,550 | 8,164,753 | 89.54 |
| **OE-S-Rep.3** | 9,939,527 | 882 | 9,628,084 | 8,705,262 | 90.42 |

**Supplementary Table S2.** List of differential expressed (≥ 2 fold) salt-responsive genes in AtSOAR1 expression plants compared with that of wild-type plants under NaCl treatment by GO analysis.

| Gene ID | | Gene name | | Description | Change (log2 fold) |
| --- | --- | --- | --- | --- | --- |
| Os01g0184100 | HSP18.0-CII | | Class II small heat shock protein | | -1.82 |
| Os01g0197700 | OsCKX2 | | Cytokinin oxidase/dehydrogenase | | 4.37 |
| Os01g0225600 | LEA14 | | late embryogenesis abundant protein, | | -0.87 |
| Os01g0654400 | Os01g0654400 | | Similar to Salt tolerant correlative protein | | -1.33 |
| Os01g0699100 | MAPKKK63 | | MAP kinase kinase kinase | | -1.16 |
| Os01g0705200 | WSI18/OsLEA14 | | Late embryogenesis abundant protein repeat containing protein | | -1.26 |
| Os01g0733200 | HsfC1b | | Heat shock factor, | | -1.91 |
| Os01g0839100 | OsZFP179 | | Cys2/His2-type zinc finger protein | | -1.64 |
| Os01g0867300 | OsABF1 | | bZIP transcription factor, | | -1.03 |
| Os01g0868000 | ERF99 | | AP2/ERF transcription factor | | 1.36 |
| Os01g0884300 | OsNAC6 | | NAC transcription factor | | -1.01 |
| Os02g0662000 | RCc3 | | Root-specific RCc3 protein | | -2.07 |
| Os02g0669100 | OsLEA23 | | SK3-type dehydrin | | -0.82 |
| Os03g0168100 | LEA16 | | Late embryogenesis abundant protein repeat containing protein | | -1.82 |
| Os03g0179400 | RLCK103 | | Drought-inducible receptor-like cytoplasmic kinase | | -2.01 |
| Os04g0534600 | PEX11-4 | | Peroxisomal protein | | -1.63 |
| Os04g0554500 | HYPRP6 | | Hybrid proline-rich protein | | -2.39 |
| Os04g0560600 | CDPK12 | | Calcium-dependent protein kinase | | 2.59 |
| Os04g0648900 | ERF8 | | Similar to Dehydration responsive element binding protein 2F (DREB2F protein) | | -1.40 |
| Os04g0659300 | OsRMC | | Receptor-like protein | | -1.77 |
| Os05g0111300 | OsMT2b | | Metallothionein, Reactive oxygen species (ROS) scavenger protein | | 2.64 |
| Os05g0381400 | AWPM-19 | | AWPM-19-like protein | | -1.98 |
| Os05g0572700 | ABI1 | | Similar to protein phosphatase 2C ABI1 | | -1.15 |
| Os07g0500300 | OsGAP | | C2-domain abscisic acid-related protein, CAR protein | | 1.01 |
| Os08g0537900 | OsERF106 | | ethylene-responsive factor | | -1.41 |
| Os08g0546800 | HSFB2B | | Class B heat shock factor | | -1.40 |
| Os10g0419300 | HsfA6a | | Heat stress transcription factor | | -0.77 |
| Os10g0529500 | OsGSTU30 | | Tau class glutathione S-transferase | | 0.85 |
| Os11g0117600 | WRKY50 | | WRKY transcription factor | | 2.20 |
| Os11g0211800 | OsDT11 | | Cysteine-rich peptide | | 2.40 |
| Os11g0592200 | PR4 | | Pathogenesis-related (PR) protein, | | 0.92 |
| Os12g0435200 | NCED2 | | Carotenoid-cleavage dioxygenase | | 1.08 |
| Os12g0555000 | RSOSPR10 | | Root-specific pathogenesis-related (PR) protein | | 0.80 |

**Supplementary Table S3.** PCR primers used in this study.

1. **Primers for generating the transgenic lines**

| Primer Name | Sequence（5'-3'） |
| --- | --- |
| OE-AtSOAR1-F | CGGGATCCATGAACTCTCTGTTCACCGC |
| OE-AtSOAR1-R | GGGGTACCCTCAAAATCCCCTGCAT |
| OE-OsSOAR1-F | TTAATTAAATGCTGGCCAGGCCTGGGCG |
| OE-OsSOAR1-R | GGCGCGCCAAGAAAGAGGTGCCATTACAGAG |

1. **Primers for RT-PCR**

| Primer Name | Sequence（5'-3'） |
| --- | --- |
| RT-Os01g0889200-F | ATGTGAATCTTCCATCCATCCAG |
| RT-Os01g0889200-R | CATTTTGCCCAAATATCCTTGTG |
| RT-Os12g0532500-F | ACTCTTGCAGGTGGATGCACAT |
| RT-Os12g0532500-R | GTCATCAATTGTGGTGTGGTCG |
| RT-Os08g0220600-F | CGAAACAAACAATAATCTGGAAGCG |
| RT-Os08g0220600-R | TGTGAACAGCCAAAAGGCAAAA |
| RT-Os07g0418000-F | CGAAACAAACAATAATCTGGAAGCG |
| RT-Os07g0418000-R | TGTGAACAGCCAAAAGGCAAAA |

1. **Primers for qPCR**

| Primer Name | Sequence（5'-3'） |
| --- | --- |
| qPCR-Actin1-F | GATGACCCAGATCATGTTTG |
| qPCR-Actin1-R | GGGCGATGTAGGAAAGC |
| qPCR-AtSOAR1-F | TACGGAACTTAGGTTGCTTGAG |
| qPCR-AtSOAR1-R | AACAACAGTCGGCTTCACATTC |
| qPCR-OsSOAR1-F | GAGCGGGCTTACAATATCCTTCTC |
| qPCR-OsSOAR1-R | CAAGAGCGTACACAATGGGGTT |
| qPCR-ABI3-F | CCCAACAACAAAAGCAGGAT |
| qPCR-ABI3-R | CCTTTGTATTGGACGAGACG |
| qPCR-RAB16A-F | CAGCTCAAGCTCGTCTGA |
| qPCR-RAB16A-R | GCTTCTCCTTGATCTTCTCCTT |
| qPCR-RAB21-F | CACACCACAGCAAGAGCTAAGTG |
| qPCR-RAB21-R | TGGTGCTCCATCCTGCTTAAG |
| qPCR-OsWRKY50-F | GGGTATCAATGGAGGAAGTATGGTC |
| qPCR-OsWRKY50-R | GGTTGTTGGTAGTACGGACGGT |
| qPCR-OsNAC6-F | GAGATGAGGACTCGAGAAAGGC |
| qPCR-OsNAC6-R | GTAATAATTAACCAATGAAGAAACAGAAACAG |
| qPCR-ERF8-F | ATAAGAACAAGGGAAGGGCAGC |
| qPCR-ERF8-R | GGAGAAGAGAGAGAGGAAAAAAAAACA |
| qPCR-Os01g0228400-F | GTTAGAGGAGGCCATCTTGTGTTGT |
| qPCR-Os01g0228400-R | ACATAAACATATTTCCCAGTCTTCCTTC |
| qPCR-Os06g0216400-F | GGAGCTTTTCGATTGTATGGGC |
| qPCR-Os06g0216400-R | CGTCTGTTGCTGCACTGTCTGT |
| qPCR-Os02g0170000-F | TGAAAGGAAGAACAGGAAATACAACG |
| qPCR-Os02g0170000-R | CGACATTCACAAGATCATCAAAAAGA |
| qPCR-Os02g0697500-F | GCCGGCGACTACATAATCCTCT |
| qPCR-Os02g0697500-R | ACTCCATCTGGTGCAGCATCTC |
| qPCR-Os06g0184866-F | ATGCTTTTCTCTACTACTCCACTCTACTTGTC |
| qPCR-Os06g0184866-R | CACTCCTCCTGCTTCTTCTCCTC |

1. **Primers for semi-quantitative qPCR**

| Primer Name | Sequence（5'-3'） |
| --- | --- |
| RT-Actin1-F | GATGACCCAGATCATGTTTG |
| RT-Actin1-R | GGGCGATGTAGGAAAGC |
| RT-OsSOAR1-F | CATCCACCGTCCTCTCCATACTC |
| RT-OsSOAR1-R | AATGCCCCGTTCTGTCATCAC |
| RT-ABI3-F | TCCAGAATCCCCTATCAAACAAGC |
| RT-ABI3-R | GAGTAGATCACAATGAAATCACCCTCCT |
| RT-RAB16A-F | CTCCAGCTCAAGCTCGTCTGA |
| RT-RAB16A-R | TTGTCCATGATGCCCTTCTTCT |
| RT-RAB21-F | CACCTTCACCTGCTTGCACA |
| RT-RAB21-R | GCCATCACACATTCACACTCATC |
| RT-OsWRKY50-F | TCAAGGCAGCCAAAGAGTAAAAGG |
| RT-OsWRKY50-R | GATGTGCATGGAGTAGGGAAGGTC |
| RT-OsNAC6-F | ACGAGGAGCTGGTGATGCACTAC |
| RT-OsNAC6-R | CTGAGGCTGTTCTTCTTGCGG |
| RT-ERF8-F | CTGCTTCTGCTGCTGCTGCT |
| RT-ERF8-R | CGACCTCGATGGCCTTCTTC |
| RT-Os01g0228400-F | GTTAGAGGAGGCCATCTTGTGTTGT |
| RT-Os01g0228400-R | ACATAAACATATTTCCCAGTCTTCCTTC |
| RT-Os06g0216400-F | GGCTGCTCTTTCTCCTCTTCCC |
| RT-Os06g0216400-R | CATCTCCACATGATCCCACCTCT |
| RT-Os02g0170000-F | CATTCCTTCCACACACAATCTTTT |
| RT-Os02g0170000-R | TTTTCGGTACATCTTGCCTTCA |
| RT-Os02g0697500-F | GCCGGCGACTACATAATCCTCT |
| RT-Os02g0697500-R | ACTCCATCTGGTGCAGCATCTC |
| RT-Os06g0184866-F | CTACTCCACTCTACTTGTCACGTGTTTTG |
| RT-Os06g0184866-R | CCTCCTGCTTCTTCTCCTCCAG |
